# Risk factors for recurrence after Bankart repair: a systematic review and meta-analysis

**DOI:** 10.1186/s13018-022-03011-w

**Published:** 2022-02-20

**Authors:** Mingtao Zhang, Jiaxin Liu, Yaofei Jia, Guangrui Zhang, Jianping Zhou, Ding Wu, Jin Jiang, Xiangdong Yun

**Affiliations:** 1 Department of Orthopaedics, Lanzhou University Second Hospital, No. 82 Cuiyingmen, Chengguan District, Lanzhou, 730030 Gansu China; 2 People’s Hospital of Changwu County, Xianyang, 713600 Shanxi People’s Republic of China

**Keywords:** Anterior shoulder, Instability, Bankart repair, Recurrence, Risk factors, Meta-analysis

## Abstract

**Background:**

The aim of this literature review was to identify preoperative risk factors associated with recurrent instability after Bankart repair.

**Methods:**

The PubMed, Web of Science, Embase, and Cochrane Library databases were searched for potentially eligible articles. Two reviewers independently screened the titles and abstracts using prespecified criteria. Articles were included if they clearly stated the risk factors for recurrence after Bankart repair. Data on patient characteristics and recurrence rate were collected from each study. A random-effects model was used for the meta-analysis and the statistical analysis was performed using Review Manager 5.4 software.

**Results:**

Nineteen studies that included 2922 participants met the inclusion criteria. The overall pooled prevalence of recurrent instability was 15.3% (range 6.9–42). The mean follow-up duration was 40.5 months (18–108). Twenty-one risk factors were identified, 10 of which were explored quantitatively. Statistically significant risk factors for recurrent instability following a Bankart procedure were age under 20 years (odds ratio [OR] 4.24, 95% confidence interval [CI] 2.8–96.23, *p* < 0.00001), a Hill-Sachs lesion (OR 3.61, 95% CI 2.06–6.33, *p* < 0.00001), a glenoid bone lesion (OR 2.8, 95% CI 1.96–4.01, *p* < 0.00001), shoulder hyperlaxity (OR 4.55, 95% CI 2.19–9.44, *p* < 0.0001), and an off-track lesion (OR 5.53, 95% CI 2.21–13.86, *p* = 0.0003). There was moderate evidence indicating that male sex (OR 1.6, 95% CI 1.07–2.37, *p* = 0.02) and playing contact sports (OR 1.54, 95% CI 0.96–2.45, *p* = 0.07) were further risk factors. Dominant side, a superior labrum from anterior to posterior (SLAP) lesion, and more than five preoperative dislocations were not found to be risk factors.

**Conclusions:**

Patients younger than 20 years of age, a Hill–Sachs lesion, a glenoid bone lesion, shoulder hyperlaxity, and an off-track lesion appear to be significant predictors of recurrent instability following a Bankart procedure. Factors such as male sex and playing contact sports were associated with recurrent instability. Dominant side, a SLAP lesion, and more than five preoperative dislocations were not significant risk factors.

**Supplementary Information:**

The online version contains supplementary material available at 10.1186/s13018-022-03011-w.

## Background

Traumatic anterior shoulder instability is a common injury that accounts for 50% of all human joint dislocations and is most often associated with playing sports and road traffic accidents [1]. Shoulder dislocation usually occurs on the anterior side because the articular surface faces the anterior lateral side and the anterior joint capsule is weaker [2]. When anterior shoulder instability progresses to recurrent dislocation, the financial and psychological burden can be substantial.

The treatment of anterior shoulder instability mainly includes conservative and surgical treatments. As surgical treatment is thought to provide better stabilization, anterior shoulder instability is usually treated surgically. Various open and arthroscopic techniques have been developed to address instability of the glenohumeral joint. In patients with glenoid bone lesion greater than 20–25%, bone block procedures are indicated, including the Bristow, Latarjet, and Eden–Hybinette procedures [3, 4]. Although bone block procedures can reduce postoperative recurrence rates, it is associated with more complications [5]. In addition, when the glenoid bone loss is lesser than 20%, the Bankart repair is a viable corrective procedure for anterior instability. With the rapid development of arthroscopic techniques for shoulder surgery, the arthroscopic Bankart procedure is now widely used. Compared with an open Bankert procedure, the arthroscopic approach has several advantages, including a lower complication rate, better diagnostic ability, less risk of stiffness and postoperative pain, and the overall medium to long term clinical results are satisfactory [6–8].

However, recent studies have shown recurrence rates of 4%–19% after arthroscopic Bankart repair [8–11]. Patients with failed Bankart repairs can be treated by procedures such as open or arthroscopic Bankart and Latarjet procedures [12, 13]. Numerous factors affect a good outcome after a Bankart procedure, such as sex, age at the time of operation, presence of a glenoid defect, a Hill-Sachs lesion, number of dislocations before initial surgery, shoulder hyperlaxity, number of anchors, and a bony Bankart lesion [14, 15]. Porcellini et al. [16] found a possible association of recurrent instability with male sex, and also other studies reported that male sex is a risk factor for recurrence after primary traumatic shoulder instability [17, 18], while other researchers have not found any association [11, 19–21]. There is ongoing controversy regarding the relationship between recurrent rates and glenoid bone lesions. Shigeto et al. [22] reported that patients with a Hill-Sachs lesion were more prone to recurrent instability. In contrast, Shibata et al. [23] and Van et al. [21] concluded that there was no relationship between this type of lesion and recurrent instability. Su et al. [24] reported that 10 (37%) of 27 patients who experienced recurrent instability had shoulder hyperlaxity. Similarly, Shin et al. [25] found that 58% of patients with recurrent instability had signs of hyperlaxity. However, other studies have demonstrated that shoulder hyperlaxity had no association with recurrent instability [15, 26]. It is generally accepted that there is a need to identify risk factors for recurrent shoulder instability after a Bankart procedure that are modifiable. However, most of the evidence regarding these risk factors is based on clinical opinion or the findings of cross-sectional studies.

Therefore, this systematic review and meta-analysis aimed to identify risk factors associated with recurrent shoulder instability within at least one year following Bankart arthroscopic in the hope of improving preoperative recognition of patients at risk of failure.

## Methods

This review and meta-analysis was performed in accordance with the PRISMA (Preferred Reporting Items for Systematic Reviews and Meta-Analyses) guidelines [27]. PRISMA checklist were showed in Additional file 1.

### Literature search

We consulted an independent information technology (IT) specialist during the designing phase of the search process. The developed search strategy is shown in Table 1. The PubMed, Embase, Web of Science, and Cochrane Library databases were searched till June 2020, for all relevant publications written in English using the following search terms: (“shoulder instability” OR “shoulder dislocation” OR “Bankart”) AND (“recurrent” OR “recurrence” OR “redislocation”) AND (“risk” OR “factor”). Potentially relevant articles were identified by screening titles and abstracts. The full-text versions of articles that met the inclusion criteria were obtained. The reference lists for the included articles were searched to identify further relevant studies.

**Table 1:** Search strategy keywords

Concept	Keywords used in the strategy
Shoulder	Shoulder* OR glenohumeral* OR GHJ
Bankart	Bankart
Dislication and instibility	Instabilit* OR unstable OR subluxat* OR stabil* OR stabl*OR luxat* OR disarticulat* OR detach* OR disassociat*disengage* OR sublux* OR dislocat*
Recurrent	Recurr* OR reocurr* OR redislocat* OR repeat*
Risk	Risk* OR factor* OR prevalen* OR predict* OR incidence* OR “odds ratio”

*Truncation of search term

### Inclusion and exclusion criteria

Studies that met the following inclusion criteria were reviewed: (1) clinical trial investigating patients with anterior shoulder instability treated by Bankart repair; (2) subluxation or dislocation confirmed by radiological evidence or clinical testing and recurrence rate recorded as an outcome measure; (3) a follow-up duration of at least one year; and (4) publication in English. The exclusion criteria were as follows: (1) study population that included patients with multidirectional or posterior shoulder instability; (2) papers published as case reports, reviews, meta-analyses, technical notes, biomechanical studies, and abstract only; and (3) studies with missing clinical outcomes data.

### Assessment of study quality

Two reviewers evaluated the quality of all included articles using the Newcastle–Ottawa Scale (NOS) checklist. The NOS rates studies according to patient selection, comparability between groups, and ascertainment of outcome of interest. This scale assigns a specific score to each article based on the quality of the research. The score ranges from 0 to 9 stars (1–3, poor; 4–6, intermediate; 7–9, high). Disagreements between the reviewers were resolved by consensus after discussion.

### Data extraction

Two reviewers independently selected suitable articles for full-text review by screening all titles and abstracts. Endnote X9 was used to review and extract data, including authors, publication year, sample size, patient demographics, study design, and recurrent instability rate. When data were not provided, the authors were contacted directly. When authors could not provide the data, the study was excluded.

### Statistical analysis

Studies that reported rates of recurrent anterior shoulder instability after Bankart repair were subjected to meta-analysis. Studies for which calculation of an odds ratio (OR) was possible were included in the meta-analysis. Heterogeneity was assessed using the *I*^2^ statistic. A fixed-effects mode was used to analyze the data when the *I*^2^ value was ≤ 50% (indicating slight statistical heterogeneity between studies); otherwise, a random-effects model was used. Estimated rates were assessed as pooled proportions with the 95% confidence interval (CI). The statistical analysis and generation of figures were performed using Review Manager version 5.4 software.

## Results

### Search results

**Fig. 1:**
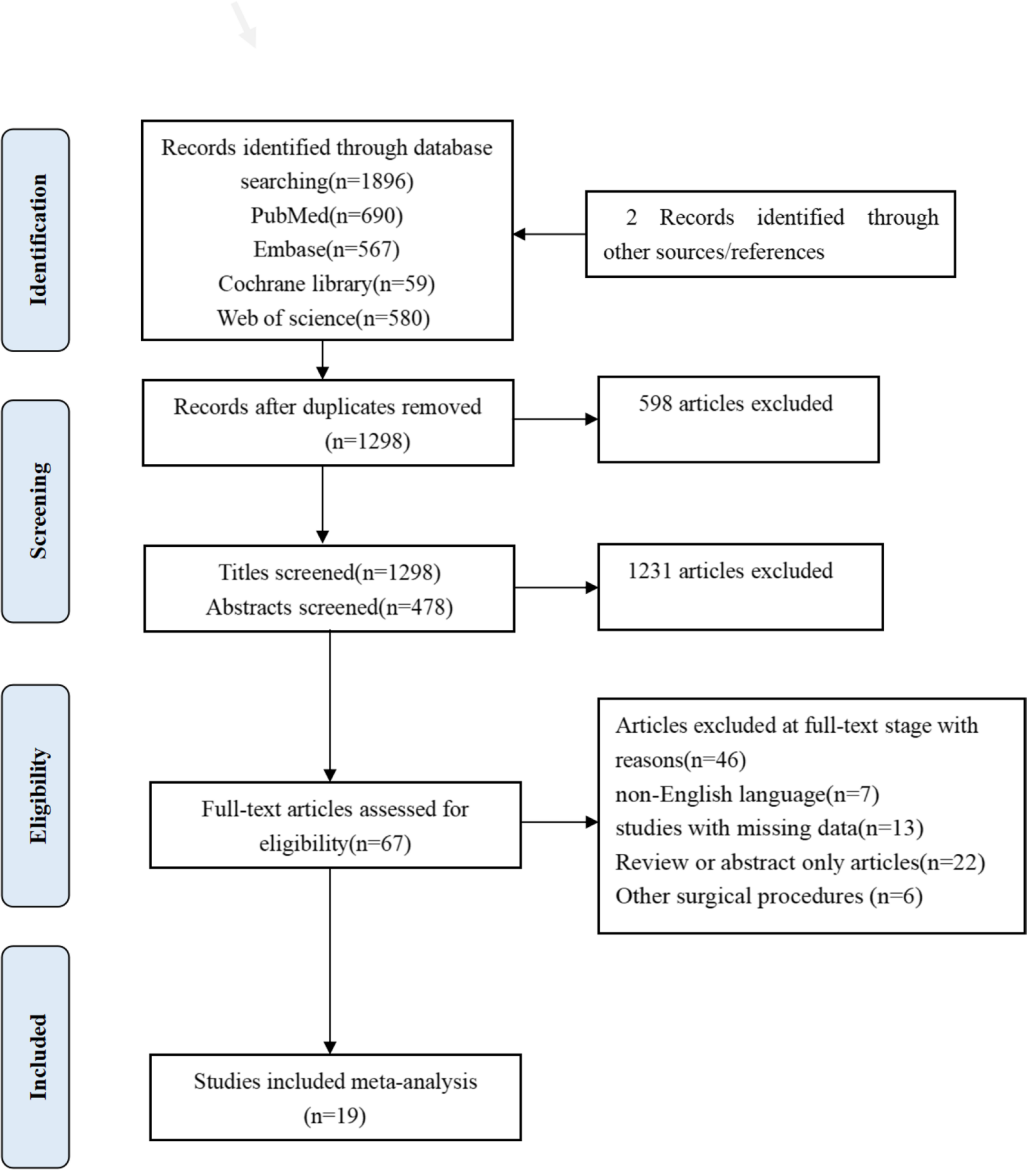
Search result

A total of 1896 studies were identified for review. An additional two articles were found by manually searching reference lists. Sixty-seven articles were potentially relevant after screening of titles and abstracts (Fig. 1). Nineteen articles met the inclusion criteria and were published in full-text form (Table 2).

**Table 2:** Characteristics of included studies

Lead author (year)	Location	No. of shoulders	Age (range)	Gender (male%)	Follow-up (months)	Total recurrence	Total recurrence (%)	Study design	NOS
Ungersbock(1995)	Switzerland	42	19–57 years	75.0	47	4	9.5	Retrospective	6
Hayashida(1998)	Japan	82	13–50 years	76.8	40	13	18.0	Retrospective	6
Tamali(1999)	Japan	87	15–60 years	82.8	18	21	24.0	Retrospective	5
Porcellini (2009)	Italy	385	NR	72.2	36	31	8.1	Prospective	7
Flinkkila(2010)	Finland	174	15–58 years	71.8	51	33	19.0	Retrospective	6
Shibata(2014)	Japan	102	14–40 years	79.0	67	9	8.8	Retrospective	8
Locher(2016)	Germany	254	15–45 years	NR	22	29	11.4	Retrospective	6
Nakagawa(2017)	Japan	296	NR	83.3	24	42	16.3	Prospetive	6
Shigeto(2017)	Japan	113	NR	89.4	24	23	20.4	Retrospective	6
Pogorzelski(2018)	USA	72	17–33 years	72.2	24	10	13.9	Retrospective	5
Su(2018)	USA	65	15–57 years	67.7	56	27	42.0	Retrospective	7
Dekker(2020)	USA	405	18–47 years	88.9	61	60	14.8	Retrospective	8
Boileau(2007)	France	131	14–62 years	78.6	31	19	14.5	Prospective	7
Burkhart(2000)	USA	194	15–64 years	87.6	27	21	10.8	Retrospective	7
Thal(2007)	USA	72	15–64 years	79.2	24	5	6.9	Retrospective	7
Voos(2009)	USA	73	15–55 years	83.6	33	13	18.0	Retrospective	7
van(2011)	Netherlands	68	19–56 years	66.2	108	24	35.0	Retrospective	6
Kandziora(2000)	Germany	163	14–52 years	79.8	46	44	24.4	Retrospective	8
Imhoff (2010)	Germany	190	14–59 years	73.7	37	27	14.2	Retrospective	7

*NR* not reported

### Characteristics of included studies

The 19 eligible studies included 2,968 shoulders in patients aged 12–64 years. The total recurrence rate was 15.3% (454/2968) during a mean follow-up of 40.5 months (range 18–108). Sixteen (84.2%) of the 19 studies were retrospective, three (15.8%) were prospective, three had a case–control design, four were cohort studies, and 12 were reported as case series. The studies had a mean NOS quality score of 6.6 ± 0.9 (Table 2). Three studies were of high quality and 14 were of intermediate quality. Two studies (by Tamali et al. [28] and Pogorzelski et al. [29]) were of low quality but had clearly defined follow-up durations (Table 2).

### Risk factors

Twenty-one common risk factors for recurrent shoulder instability after a Bankart procedure were identified in the 21 included articles. Risk factors identified in more than two studies included age, sex, type of sport, shoulder hyperlaxity, dominant side, number of preoperative dislocations, a SLAP tear, an off-track lesion, a Hill-Sachs lesion, and a glenoid bone lesion. Other risk factors related recurrent shoulder instability were showed in Additional file 2.

### Patient-related factors

#### Age

**Fig. 2:**
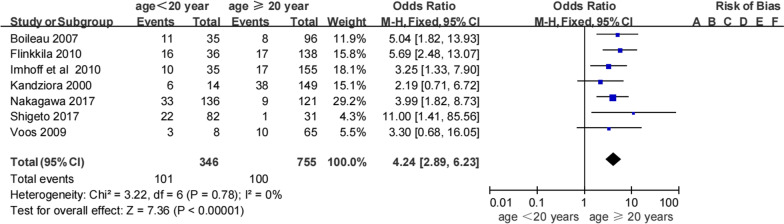
Age as a risk factor

Seven studies [11, 22, 30–34] reported an association between age and recurrent instability (Table 3). Some studies had grouped data for patients aged ≥ 20 years and were unable to provide raw data; these data were grouped into two age brackets, namely, ≤ 19 years and ≥ 20 years. Pooled data showed that 29.1% (101/346) of patients aged ≤ 19 years experienced an instability event following the Bankart procedure and 13.2% (100/755) aged ≥ 20 years experienced recurrent instability (Table 3). Meta-analysis revealed that patients aged ≤ 19 years were more likely to experience recurrent instability than those aged ≥ 20 years (OR 4.24, 95% CI 2.89–6.23, *Z* = 7.36, *p* < 0.00001, *I*^2^ = 0%; Fig. 2). Based on these studies, we found that age younger than 20 years was an important risk factor for recurrent instability following a Bankart procedure.

**Table 3:** Recurrent shoulder instability in people aged under 20 years, compared with 20 years and older

Age	Imhoff et al.		Flinkkila et al.		Kandziora et al.		Nakagawa et al.		Shigeto et al.		Voos et al.		Boileau et al.		Total	
	Rec	Non	Rec	Non	Rec	Non	Rec	Non	Rec	Non	Rec	Non	Rec	Non	Recurrence	Non-recurrence
< 20 years	10	25	16	20	6	8	33	103	22	60	3	5	11	24	29.1% (101/346)	70.8% (245/346)
≥ 20 years	17	138	17	121	38	111	9	112	1	30	10	55	8	88	13.2% (100/755)	86.7% (655/755)
Total	27	163	33	141	44	119	42	215	23	90	13	60	19	112	18.3% (201/1101)	81.7% (900/1101)

Non, no shoulder instability; Rec, recurrent shoulder instability

#### Sex

**Fig. 3:**
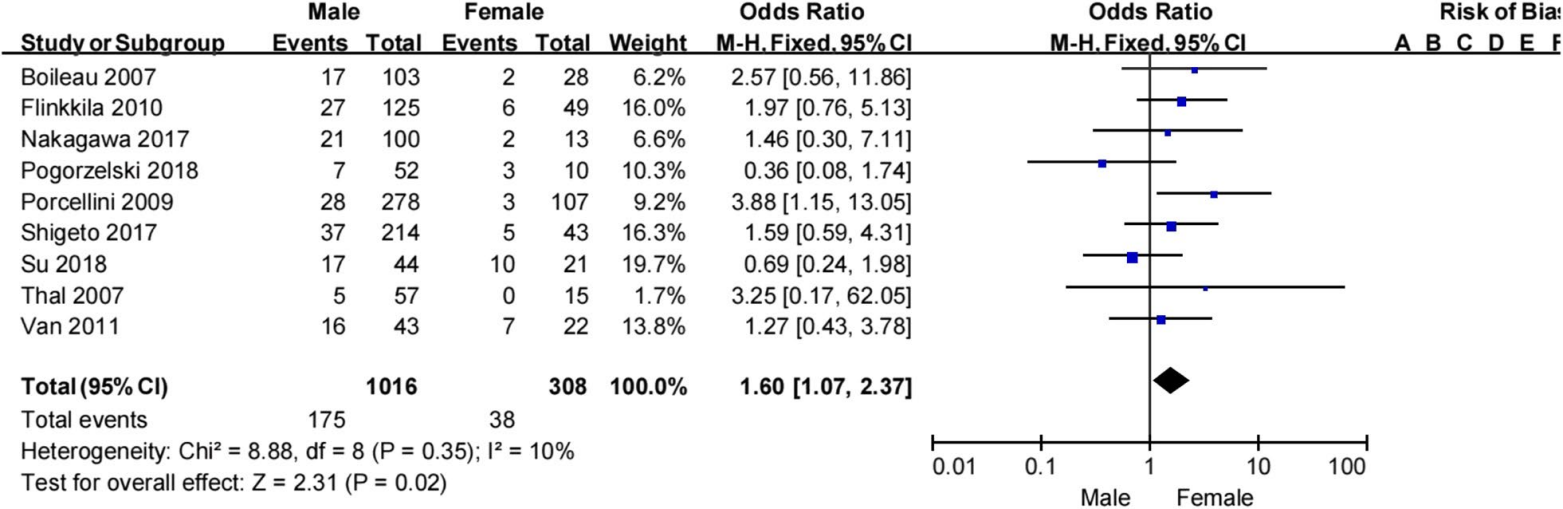
Sex and recurrent shoulder instability

Nine studies [16, 20–22, 24, 29–31, 34] investigated the effect of patient sex on the risk of recurrent instability following a Bankart procedure and found an overall recurrence rate of 17.2% in men and 12.3% in women (Table 4). Meta-analysis showed that women were more likely to experience recurrent instability than women (OR 1.6, 95% CI 1.07–2.37, *Z* = 2.31, *p* = 0.02, *I*^2^ 10%; Fig. 3). Therefore, there was moderate evidence to suggest that male sex is an important risk factor for recurrent instability following Bankart repair.

**Table 4:** Sex and recurrent shoulder instability

Sex	Porcellini et al.		Flinkkila et al.		Nakagawa et al.		Shigeto et al.		Pogorzelski et al.		Su et al.		Boileau et al.		Thal et al.		Van et al.		Total	
	Rec	Non	Rec	Non	Rec	Non	Rec	Non	Rec	Non	Rec	Non	Rec	Non	Rec	Non	Rec	Non	Rec	Non
Male	28	250	27	98	37	177	21	79	7	45	17	27	17	86	5	52	16	27	17.2%(175/1016)	82.7%(841/1016)
Fmale	3	104	6	43	5	38	2	11	3	7	10	11	2	26	0	15	7	15	12.3%(38/308)	87.6%(270/308)
Total	31	354	33	141	42	215	23	90	10	52	27	38	19	112	5	67	23	42	16.0% (213/1324)	83.9% (1111/1324)

Non, no shoulder instability; Rec, recurrent shoulder instability

#### Type of sport

**Fig. 4:**
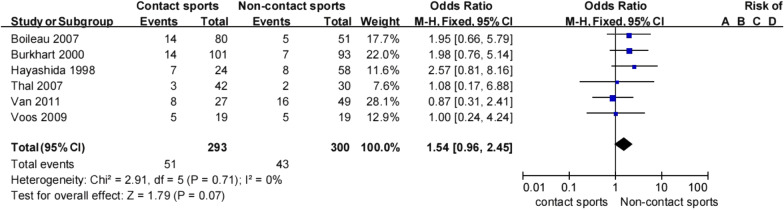
Type of sport and recurrent shoulder instability

The types of sport played by patients with recurrent shoulder instability was typically divided into contact and non-contact. Six studies [11, 20, 21, 30, 35, 36] investigated whether type of sport played was a risk factor for recurrent instability following a Bankart procedure. Pooled data showed that patients who played a contact sport were 1.54 times more likely to experience an instability event following a Bankart procedure than those who played a non-contact sport (17.4% [51/293] vs 14.3% [43/300]; Table 5). This finding was not statistically significant but was homogeneous (OR 1.54, 95% CI 0.96–2.45, *Z* = 1.79, *p* = 0.07, *I*^2^ 0%; Fig. 4). Therefore, there was moderate evidence to suggest that contact sport is an important risk factor for recurrent instability following a Bankart procedure.

**Table 5:** Type of sport and recurrent shoulder instability

Type of sport	Hayashida et al.		Van et al.		Boileau et al.		Burkhart et al.		Thal et al.		Voos et al.		Total	
	Rec	Non	Rec	Non	Rec	Non	Rec	Non	Rec	Non	Rec	Non	Recurrence	Non-recurrence
Contact sports	7	17	8	19	14	66	14	87	3	39	5	14	17.4% (51/293)	82.6% (242/293)
Noncontact sports	8	50	16	33	5	46	7	86	2	28	5	14	14.3% (43/300)	85.7% (257/300)
Total	15	67	24	52	19	112	21	173	5	67	10	28	15.9% (94/593)	84.1% (499/593)

Non, no shoulder instability; Rec, recurrent shoulder instability

#### Dominant side

**Fig. 5:**
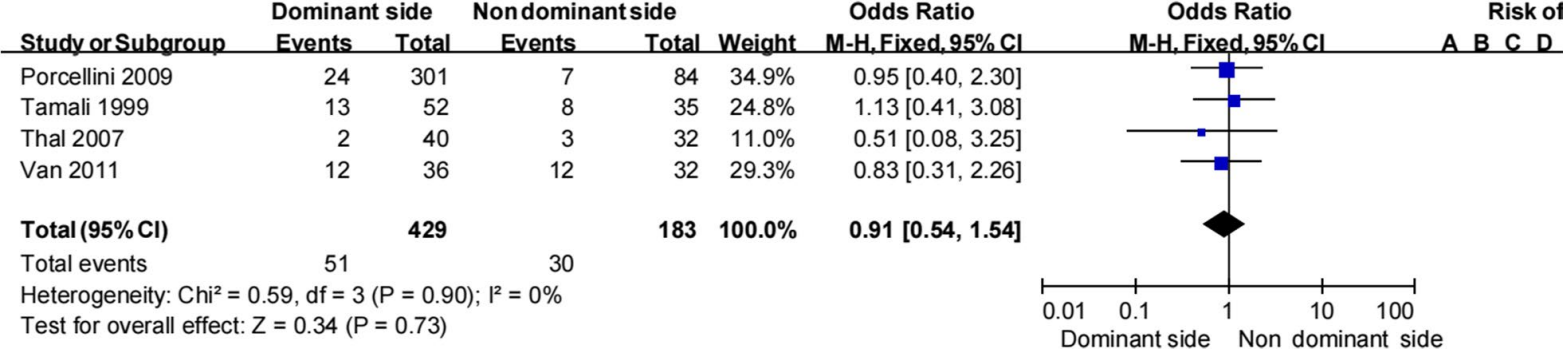
Dominant side and recurrent shoulder instability

Four studies [16, 20, 21, 28] presented information regarding side dominance of the shoulder with recurrent instability. Pooled data showed that recurrent instability after a Bankart procedure was less common on the dominant side than on the non-dominant side (11.9% [51/429] vs 16.4% [30/183]; Table 6). This result was not statistically significant but was homogeneous (OR 0.91, 95% CI 0.54–1.54, *Z* = 0.34, *p* = 0.73, *I*^2^ = 0%; Fig. 5). Therefore, there is no evidence to suggest that dominant side is an important risk factor for recurrent instability following a Bankart procedure.

**Table 6:** Dominant side and recurrent shoulder instability

	Tamali et al.		Porcellini et al.		Thal et al.		Van et al.		Total	
	Rec	Non	Rec	Non	Rec	Non	Rec	Non	Rec	Non
Dominant side	13	39	24	277	2	38	12	24	11.9% (51/429)	88.1% (378/429)
Non-dominant side	8	27	7	77	3	29	12	20	16.4% (30/183)	83.6% (153/183)
Total	21	66	31	354	5	67	24	44	13.2% (81/612)	86.8% (531/612)

Non, no shoulder instability; Rec, recurrent shoulder instability

#### Number of preoperative dislocations

**Fig. 6:**
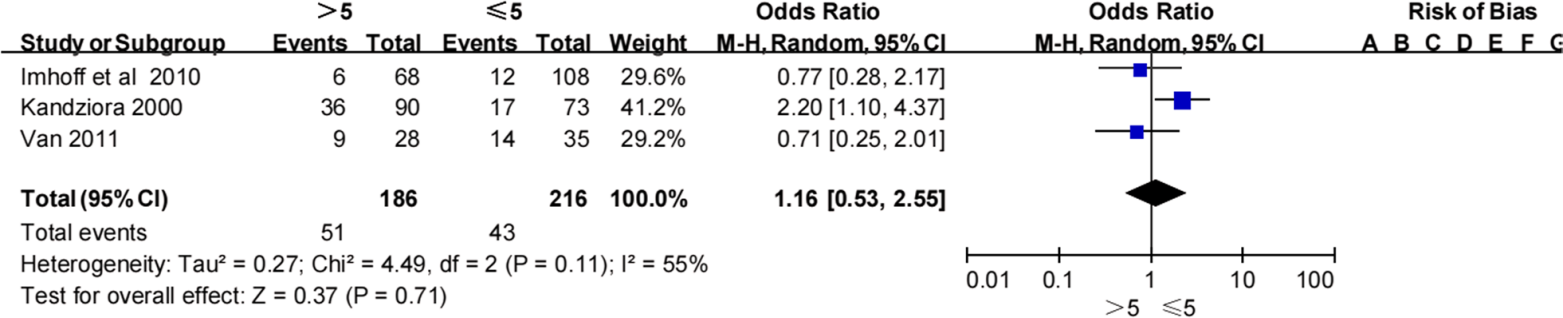
Number of preoperative dislication and recurrent shoulder instability

Three studies [20, 21, 33] investigated the association between number of preoperative dislocations (more than five) and recurrent instability following Bankart repair (Table 7). Pooled data indicated that patients with more than five preoperative dislocations experienced a higher rate of recurrent instability than those with fewer dislocations (27.4% [51/186] vs 20% [43/216]). More than five dislocations was associated with a significantly higher odds of recurrent instability (OR 1.16, 95% CI 0.53–2.55, *Z* = 0.37, *p* = 0.71, *I*^2^ = 55%; Fig. 6). Therefore, we found that there was marginal to no evidence to suggest that more than five preoperative dislocations is an important risk factor for recurrent instability following a Bankart procedure.

**Table 7:** Number of preoperative dislocation and recurrent shoulder instability

No. of dislocation	Kandziora et al.		Imhoff et al.		Van et al.		Total	
	Rec	Non	Rec	Non	Rec	Non	Rec	Non
≤ 5	17	56	12	96	14	21	20.0% (43/216)	80.0% (173/216)
> 5	36	54	6	62	9	19	27.4% (51/186)	72.6% (135/186)
Total	53	110	18	152	23	40	23.4% (94/402)	76.6% (308/402)

Non, no shoulder instability; Rec, recurrent shoulder instability

#### Shoulder hyperlaxity

**Fig. 7:**
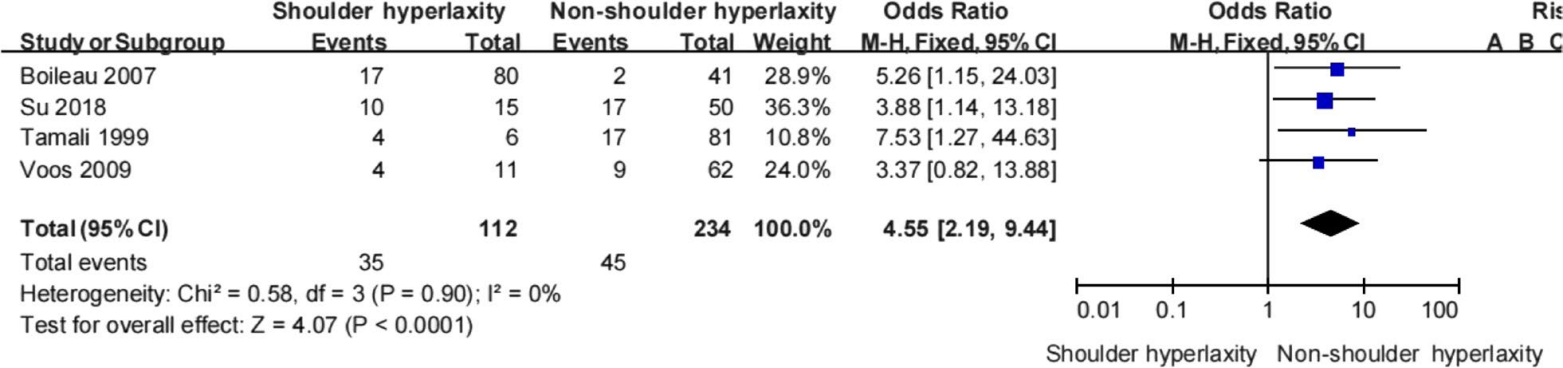
Shoulder hyperlaxity and recurrent shoulder instability

Four studies [11, 24, 28, 30] provided information on the relationship between shoulder hyperlaxity and recurrent instability. Pooled data showed that patients with shoulder hyperlaxity had a higher rate of recurrent instability than those who did not (28.7% vs 19.2%; Table 8). Moreover, patients with shoulder hyperlaxity were 4.5 times more likely to experience recurrent instability (OR 4.55, 95% CI 2.19–9.44, *Z* = 4.07, *p* < 0.0001, *I*^2^ = 0%; Fig. 7). These findings strongly suggest that shoulder hyperlaxity is an important risk factor for recurrent instability following a Bankart procedure.

**Table 8:** Shoulder hyperlaxity and recurrent shoulder instability

	Tamali et al		Boileau et al		Voos et al		Su et al		Total	
	Rec	Non	Rec	Non	Rec	Non	Rec	Non	Rec	Non
Shoulder hyperlaxity	4	2	17	73	4	7	10	5	28.7% (35/122)	71.3% (87/122)
Non-shoulder hyperlaxity	17	64	2	39	9	53	17	33	19.2% (45/234)	80.8% (189/234)
Total	21	66	19	112	13	60	27	38	22.5% (80/356)	77.5% (276/356)

Non, no shoulder instability; Rec, recurrent shoulder instability

### Pathoanatomical factors

#### Hill-Sachs lesions

**Fig. 8:**
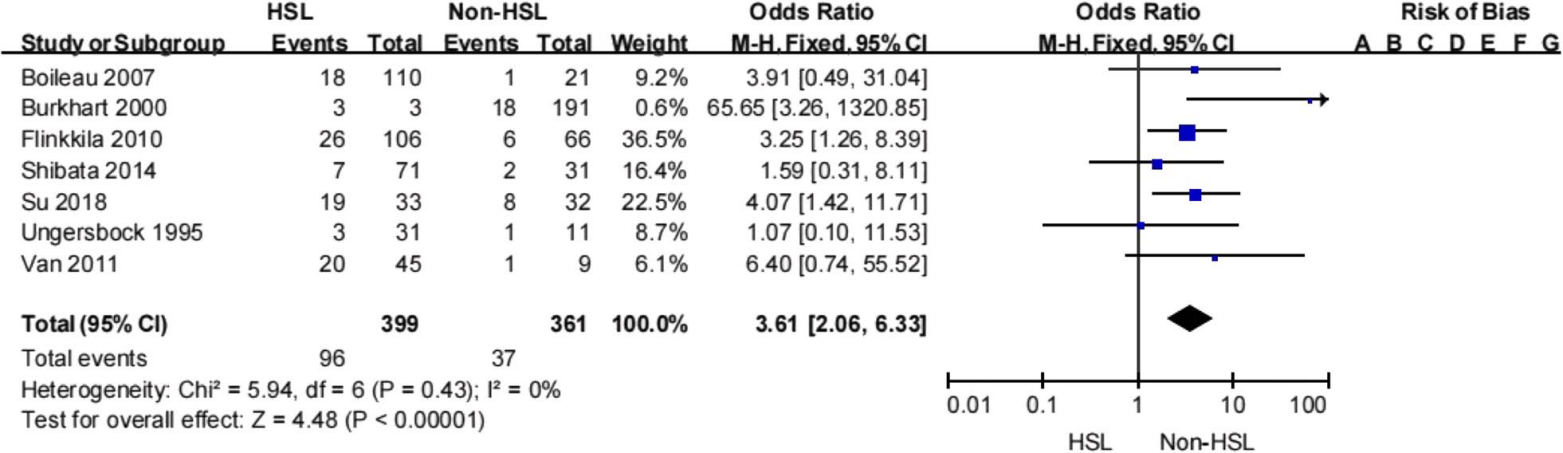
Hill-Sachs lesion and recurrent shoulder instability (HSL, Hill-Sachs lesion)

Seven studies reported on the association between presence of a Hill-Sachs lesion and recurrent instability [21, 22, 24, 30, 31, 35, 37]. When the data were combined, recurrent instability events after Bankart repair were more common in patients with radiographic evidence of a Hill-Sachs lesion than in those without this lesion (24% [96/399] vs 10.2% [37/361]; Table 9). Meta-analysis showed that patients with a Hill-Sachs lesion were more likely to experience recurrent instability (OR 3.61, 95% CI 2.06–6.33, *Z* = 4.48, *p* < 0.00001, *I*^2^ = 0%; Fig. 8). Therefore, there was strong evidence to suggest that a Hill-Sachs lesion is an important risk factor for recurrent instability following a Bankart procedure.

**Table 9:** Hill-Sachs lesion and recurrent shoulder instability

	Flinkkila et al.		Shibata et al.		Su et al.		Boileau et al.		Burkhart et al.		Ungersbock et al.		van et al.		Total	
	Rec	Non	Rec	Non	Rec	Non	Rec	Non	Rec	Non	Rec	Non	Rec	Non	Rec	Non
HSL	26	80	7	64	19	14	18	92	3	0	3	28	20	25	24.0% (96/399)	75.9% (303/399)
Non HSL	6	60	2	29	8	24	1	20	18	173	1	10	1	8	10.2% (37/361)	89.7% (324/361)
Total	32	140	9	93	27	38	19	112	21	173	4	38	21	33	17.5% (133/760)	82.5% (627/760)

HSL, Hill-Sachs lesion; Non, no shoulder instability; Rec, recurrent shoulder instability

#### Off-track lesions

**Fig. 9:**
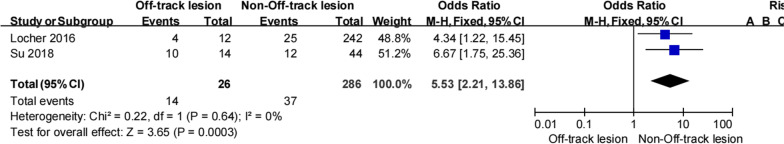
Off-track lesion and recurrent shoulder instability

Radiographic evidence of an off-track lesion was reported by two studies [24, 26]. When the data were combined, patients with an off-track lesion were markedly more likely to experience a recurrent instability event (53.8% [14/26] vs 13% [37/286]; Table 10). Pooled analysis revealed that having an off-track lesion was significantly associated with an increased likelihood of having a recurrent instability event following a Bankart procedure (OR 5.53, 95% CI 2.21–13.86, *Z* = 3.65, *p* = 0.0003). Heterogeneity was considered unimportant (*I*^2^ = 0%; *p* = 0.64; Fig. 9). Therefore, there was strong evidence to suggest that the presence of an off-track lesion is an important risk factor for recurrent instability following Bankart repair.

**Table 10:** Off-track lesion and recurrent shoulder instability

	Su et al.		Locher et al.		Total	
	Rec	Non	Rec	Non	Rec	Non
Off-track lesion	10	4	4	8	53.8% (14/26)	46.2% (12/26)
Non	12	32	25	217	13.0% (37/286)	87.0% (249/286)
Total	22	36	29	225	16.3% (51/312)	83.7% (261/312)

Non, no shoulder instability; Rec, recurrent shoulder instability

#### Glenoid bone lesions

**Fig. 10:**
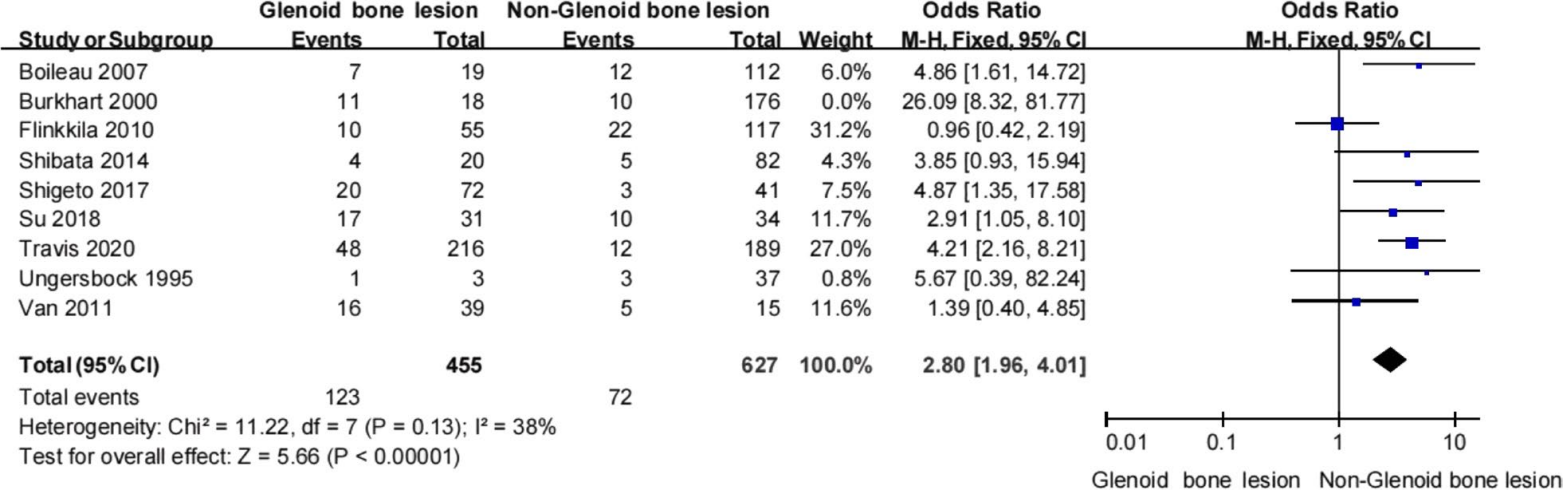
Glenoid bone lesion and recurrent shoulder instability

Nine studies [14, 21–24, 30, 31, 35, 37] reported on the recurrence rates for shoulder instability according to whether or not a glenoid bone lesion was present. Pooled data showed that patients were more likely to experience a recurrent instability event following Bankart procedure if they had a glenoid bone lesion (28.3% [134/473] vs 10.2% [82/803]; Table 11). Pooled analysis showed that having a glenoid bone lesion conferred a significantly higher odds of developing a recurrent instability event (OR 2.8, 95% CI 1.96–4.01, *Z* = 5.66, *p* < 0.00001). Heterogeneity was considered unimportant (*I*^2^ = 38%, *p* = 0.13; Fig. 10). Therefore, there was strong evidence to suggest that having a glenoid bone lesion was an important risk factor for recurrent instability following a Bankart procedure.

**Table 11:** Glenoid bone lesion and recurrent shoulder instability

	Ungersbock et al		Nakagawa et al		Su et al		Dekker et al		Boileau et al		Burkhart et al		Flinkkila et al		Shibata et al		van et al		Total	
	Rec	Non	Rec	Non	Rec	Non	Rec	Non	Rec	Non	Rec	Non	Rec	Non	Rec	Non	Rec	Non	Rec	Non
GBL	1	2	20	52	17	14	48	168	7	12	11	7	10	45	4	16	16	23	28.3% (134/473)	71.6% (339/473)
Non-GBL	3	34	3	38	10	24	12	177	12	100	10	166	22	95	5	77	5	10	10.2% (82/803)	89.7% (721/803)
Total	4	36	23	90	27	38	60	345	19	112	21	173	32	140	9	93	21	33	16.9% (216/1276)	83.1% (1060/1276)

GBL, glenoid bone lesion; Non, no shoulder instability; Rec, recurrent shoulder instability

#### SLAP lesions

**Fig. 11:**
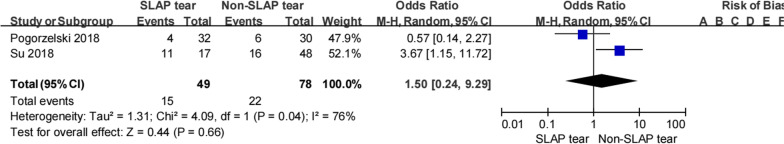
SLAP tear and recurrent shoulder instability (SLAP, superior labrum from anterior to posterior)

Two studies [24, 29] investigated the association between having a SLAP lesion and recurrent instability following a Bankart procedure (Table 12). Pooled analysis indicated that a SLAP lesion was not significantly associated with an increased likelihood of recurrent instability following a Bankart procedure (OR 1.5, 95% CI 0.24–9.29, *Z* = 0.44, *p* = 0.66). Heterogeneity was considered significant (*I*^2^ 76%, *p* = 0.04; Fig. 11). Therefore, there was no evidence to suggest that a SLAP lesion is an important risk factor for recurrent instability after Bankart repair.

**Table 12:** SLAP tear and recurrent shoulder instability

	Pogorzelski et al.		Su et al.		Total	
	Rec	Non	Rec	Non	Rec	Non
SLAP tear	4	28	11	6	30.6% (15/49)	69.4% (34/49)
Non-SLAP tear	6	24	16	32	28.2% (22/78)	71.8% (56/78)
Total	10	52	27	38	29.1% (37/127)	70.9% (90/127)

SLAP, superior labrum from anterior to posterior; Non, no shoulder instability; Rec, recurrent shoulder instability

## Discussion

This systematic review and meta-analysis yielded three main findings. First, there was strong evidence that recurrent instability following a Bankart procedure was more likely in patients younger than 20 years of age and those with a Hill-Sachs lesion, a glenoid bone lesion, shoulder hyperlaxity, or an off-track lesion. Second, there was moderate evidence that male sex and playing a contact sport was more common in patients with recurrent instability. Three, there was no evidence of recurrent involvement of the dominant side and having a SLAP lesion or more than five preoperative dislocations. In addition, this systematic review provides a quantitative analysis of the risk factors for recurrent instability after Bankart, which can help the surgeons to choose the appropriate surgical approach according to the patient and to choose bone block surgery instead of Bankart when necessary, which can reduce the rate of postoperative recurrent instability.

### Patient-related factors

Age was identified as the primary risk factor for recurrent instability following a Bankart procedure [11, 14, 16, 20, 22, 24, 30–34]. Some studies [14, 22] have suggested that patients aged ≥ 20 years have lower rates of recurrent instability following a Bankart procedure than those ≤ 19 years. Our finding that patients aged ≤ 19 years were 4.42 times more likely to experience recurrent instability than older patients is in line with this suggestion. Many factors can lead to this phenomenon, including lateral glenohumeral joint capsule insertion at a younger age, greater joint capsule elasticity at a younger age, and level of activity [38]. Another possible factor is lower compliance with postoperative rehabilitation in the younger age group. Therefore, 20 years could be used as the critical age cut-off for recurrent instability following a Bankart procedure.

There is discrepancy in the literature regarding the relationship between the recurrence rate and male sex. Procellini et al. [16] reported that 21 (90.3%) of 31 patients who experienced recurrent instability were male and three (9.7%) were female. They suggested that the risk for recurrence was approximately 3.5 times higher among male patients than female patients. However, other studies [21, 24, 30] found that sex had no effect on the likelihood of recurrent instability. Our systematic review found that men was more likely to experience recurrent instability.

There is also controversy in the literature regarding the relationship between recurrent instability and playing contact sports. Voos et al. [11] reported that 5 of 19 patients in a contact sports group experienced recurrent dislocation vs 5 of 19 in a non-contact sports group (*p* > 0.05). Thal et al. [20] found no association between playing contact sports and recurrent instability after Bankart repair. In contrast, Van et al. [21] suggested that patients who play contact sports were more prone to recurrent instability. Our meta-analysis showed that patients who played contact sports were 1.54 times more likely to experience recurrent instability than those who did not; however, this result was not statistically significant (*p* = 0.07).

Shoulder hyperlaxity is an independent risk factor for recurrence dislocation after Bankart revision and is related to the amount of plastic deformation of the capsule after recurrent instability. However, there remains controversy on this issue in the literature. Some studies [11, 24, 28, 30] found that patients with shoulder hyperlaxity had a higher rate of recurrent More than five dislocations was associated with a significantly higher odds of recurrent instability whereas Lee et al. [15] and Hayashida et al. [36] suggested that shoulder hyperlaxity has no significant effect on recurrent instability. Our meta-analysis showed that patients with shoulder hyperlaxity were 4.5 times more likely to experience recurrent instability than those without shoulder hyperlaxity (*p* < 0.0001). These findings strongly suggest that shoulder hyperlaxity is an important risk factor for recurrent instability following a Bankart procedure.

### Pathoanatomical factors

A Hill-Sachs lesion is found in 47–100% of patients who experience anterior shoulder instability [39]. The presence of a Hill-Sachs lesion on magnetic resonance imaging was found to be a strong risk factor for recurrent instability following a Bankart procedure. Some studies [23, 24, 30, 31, 35] found that patients with a Hill-Sachs lesion were more likely to experience recurrent instability whereas other studies [21, 37] found that neither the presence or magnitude of a Hill-Sachs lesion influenced the recurrent instability rate. This review found that recurrent instability events after Bankart repair were more common in patients with radiographic evidence of a Hill–Sachs lesion than in those without this lesion. Furthermore, Su et al. [24] reported that patients with an off-track lesion had a nearly ninefold increase in recurrent instability rate. Locher et al. [26] also reported that patients with an off-track lesion had an 8.3 times higher risk of recurrence that was significantly associated with failure of Bankart revision. This meta-analysis found that having an off-track lesion was significantly associated with an increased likelihood of a recurrent instability event following a Bankart procedure (*p* = 0.0003). There was strong evidence to suggest that the presence of an off-track lesion is an important risk factor for recurrent instability following Bankart repair.

The finding of decreased recurrent instability in the presence of a glenoid bone lesion was not surprising. It has been shown that a glenoid bone lesion can affect glenohumeral stability in two ways. First, the arc length of the glenoid is decreased and, second, the loss of the glenoid surface reduces the concavity of the glenoid [35, 40]. Generally, a glenoid bone lesion greater than 20–25% is considered a critical contributor to poor surgical outcomes after Bankart repair [41–43]. In a cadaveric study, Itoi et al. [43] found that a glenoid bone defect of more than 21% remained unstable after Bankart repair. Moreover, a recent study demonstrated that a glenoid bone defect of 17.3% should be considered as the critical amount of bone loss that may result in recurrent instability after arthroscopic Bankart repair [25]. Moreover, another clinical report suggested that the critical value for glenoid bone loss, especially relating to failure rates after primary arthroscopic Bankart repair for anterior shoulder instability, was 13.5% [44]. Our meta-analysis showed that a glenoid bone lesion is an important risk factor for recurrent instability following a Bankart procedure. However, there is still controversy regarding the critical amount of glenoid bone loss with regard to failure after Bankart repair. Our meta-analysis found that other factors, including dominant side, a SLAP lesion, and more than five preoperative dislocations, had no effect on recurrent instability following Bankart repair.

This review and meta-analysis has some limitations. First, it included 19 studies, of which 16 (84.2%) were retrospective and three (15.8%) were prospective. The retrospective studies were limited by incomplete information and loss to follow-up in the medical records. Therefore, our findings may be affected by the number of studies of lower quality included. Furthermore, evaluation of the heterogeneity of variables highlighted the variability among the studies. Finally, there were only three studies with a follow-up duration of more than 5 years, which may have affected our results, and we could not extract all data on bone lesions so could not analyze critical values or all types of bone lesions.

## Conclusions

This systematic review was carried out to identify the risk factors associated with recurrent shoulder instability after performing a Bankart procedure. Firstly, patients younger than 20 years of age, presence of a Hill–Sachs lesion, a glenoid bone lesion, shoulder hyperlaxity, and an off-track lesion appeared to be significant predictors of recurrent instability. Furthermore, male sex and playing contact sports were found to have an association with recurrent instability following a Bankart procedure. Finally, involvement of the dominant side, presence of a SLAP lesion, and having more than five preoperative dislocations were not significantly associated with postoperative recurrent instability. There is need for prospective cohort studies with large sample sizes that could be used in the future to confirm the value of the risk factors identified in this review.

## Supplementary Information

**Additional file 1.** PRISMA checklist of the meta-analysis.

**Additional file 2.** Other risk factors related recurrent shoulder instability.

## Data Availability

Not applicable.

## Abbreviations

CI: Confidence interval
OR: Odds ratio
SLAP: Superior labrum from anterior to posterior
PRISMA: Preferred Reporting Items for Systematic Reviews and Meta-Analyses
IT: Information technology
NOS: Newcastle–Ottawa Scale

## References

[1] Dodson CC, Cordasco FA (2008). Anterior glenohumeral joint dislocations. Orthop Clin North Am.

[2] Plath JE, Aboalata M, Seppel G, Juretzko J, Waldt S, Vogt S, Imhoff AB (2015). Prevalence of and risk factors for dislocation arthropathy: radiological long-term outcome of arthroscopic Bankart repair in 100 shoulders at an average 13-year follow-up. Am J Sports Med.

[3] Tauber M, Resch H, Forstner R, Raffl M, Schauer J (2004). Reasons for failure after surgical repair of anterior shoulder instability. J Shoulder Elbow Surg.

[4] Longo UG, Loppini M, Rizzello G, Ciuffreda M, Maffulli N, Denaro V (2014). Latarjet, Bristow, and Eden-Hybinette procedures for anterior shoulder dislocation: systematic review and quantitative synthesis of the literature. Arthroscopy.

[5] Longo UG, Loppini M, Rizzello G, Ciuffreda M, Maffulli N, Denaro V (2014). Management of primary acute anterior shoulder dislocation: systematic review and quantitative synthesis of the literature. Arthroscopy.

[6] Franceschi F, Papalia R, Del Buono A, Vasta S, Maffulli N, Denaro V (2011). Glenohumeral osteoarthritis after arthroscopic Bankart repair for anterior instability. Am J Sports Med.

[7] Adam M, Attia AK, Alhammoud A, Aldahamsheh O, Al Ateeq Al Dosari M, Ahmed G (2018). Arthroscopic Bankart repair for the acute anterior shoulder dislocation: systematic review and meta-analysis. Int Orthop.

[8] Miettinen SSA, Kiljunen T, Joukainen A (2021). Anterior glenohumeral instability treated with arthroscopic Bankart operation—a retrospective 5-year follow-up study. Orthop Traumatol Surg Res.

[9] Delgrande D, Lonjon G, Hardy P, Schoch B, Werthel JD (2021). Long-term results of arthroscopic Bankart repairs for anterior instability of the shoulder in patients aged thirty years or older. Int Orthop.

[10] García-Vega M, De La Cuadra-Virgil P, Jiménez-Cristobal J, Occhi-Gómez B, Boserman-Pérez-de Villaamil M (2021). Arthroscopic bankart repair for the management of anterior shoulder instability: mid- and long-term results. Rev Esp Cir Ortop Traumatol..

[11] Voos JE, Livermore RW, Feeley BT, Altchek DW, Williams RJ, Warren RF, Cordasco FA, Allen AA, Service HSSSM (2010). Prospective evaluation of arthroscopic Bankart repairs for anterior instability. Am J Sports Med.

[12] Franceschi F, Longo UG, Ruzzini L, Rizzello G, Maffulli N, Denaro V (2008). Arthroscopic salvage of failed arthroscopic Bankart repair: a prospective study with a minimum follow-up of 4 years. Am J Sports Med.

[13] Solomon DJ (2021). Editorial commentary: better stability found with primary Latarjet compared with those performed after a failed arthroscopic Bankart repair: should we be doing more primary Latarjet procedures?. Arthroscopy.

[14] Dekker TJ, Peebles LA, Bernhardson AS, Rosenberg SI, Murphy CP, Golijanin P, Provencher MT (2020). Risk factors for recurrence after arthroscopic instability repair-the importance of glenoid bone loss >15%, patient age, and duration of symptoms: a matched cohort analysis. Am J Sports Med.

[15] Lee SH, Lim KH, Kim JW (2018). Risk factors for recurrence of anterior-inferior instability of the shoulder after arthroscopic Bankart repair in patients younger than 30 years. Arthroscopy.

[16] Porcellini G, Campi F, Pegreffi F, Castagna A, Paladini P (2009). Predisposing factors for recurrent shoulder dislocation after arthroscopic treatment. J Bone Joint Surg Am.

[17] Robinson CM, Howes J, Murdoch H, Will E, Graham C (2006). Functional outcome and risk of recurrent instability after primary traumatic anterior shoulder dislocation in young patients. J Bone Joint Surg Am.

[18] Robinson CM, Jenkins PJ, White TO, Ker A, Will E (2008). Primary arthroscopic stabilization for a first-time anterior dislocation of the shoulder. A randomized, double-blind trial. J Bone Joint Surg Am.

[19] Sommaire C, Penz C, Clavert P, Klouche S, Hardy P, Kempf JF (2012). Recurrence after arthroscopic Bankart repair: is quantitative radiological analysis of bone loss of any predictive value?. Orthop Traumatol Surg Res.

[20] Thal R, Nofziger M, Bridges M, Kim JJ (2007). Arthroscopic Bankart repair using Knotless or BioKnotless suture anchors: 2- to 7-year results. Arthroscopy.

[21] van der Linde JA, van Kampen DA, Terwee CB, Dijksman LM, Kleinjan G, Willems WJ (2011). Long-term results after arthroscopic shoulder stabilization using suture anchors: an 8- to 10-year follow-up. Am J Sports Med.

[22] Nakagawa S, Mae T, Sato S, Okimura S, Kuroda M (2017). Risk factors for the postoperative recurrence of instability after arthroscopic Bankart repair in athletes. Orthop J Sports Med.

[23] Shibata H, Gotoh M, Mitsui Y, Kai Y, Nakamura H, Kanazawa T, Okawa T, Higuchi F, Shirahama M, Shiba N (2014). Risk factors for shoulder re-dislocation after arthroscopic Bankart repair. J Orthop Surg Res.

[24] Su F, Kowalczuk M, Ikpe S, Lee H, Sabzevari S, Lin A (2018). Risk factors for failure of arthroscopic revision anterior shoulder stabilization. J Bone Joint Surg Am.

[25] Shin JJ, Mascarenhas R, Patel AV, Yanke AB, Nicholson GP, Cole BJ, Romeo AA, Verma NN (2015). Clinical outcomes following revision anterior shoulder arthroscopic capsulolabral stabilization. Arch Orthop Trauma Surg.

[26] Locher J, Wilken F, Beitzel K, Buchmann S, Longo UG, Denaro V, Imhoff AB (2016). Hill-Sachs off-track lesions as risk factor for recurrence of instability after arthroscopic Bankart repair. Arthroscopy.

[27] Moher D, Liberati A, Tetzlaff J, Altman DG, Group P (2009). Preferred reporting items for systematic reviews and meta-analyses: the PRISMA statement. Ann Intern Med.

[28] Tamai K, Higashi A, Tanabe T, Hamada J (1999). Recurrences after the open Bankart repair: a potential risk with use of suture anchors. J Shoulder Elbow Surg.

[29] Pogorzelski J, Fritz EM, Horan MP, Katthagen JC, Provencher MT, Millett PJ (2018). Failure following arthroscopic Bankart repair for traumatic anteroinferior instability of the shoulder: is a glenoid labral articular disruption (GLAD) lesion a risk factor for recurrent instability?. J Shoulder Elbow Surg.

[30] Balg F, Boileau P (2007). The instability severity index score. A simple pre-operative score to select patients for arthroscopic or open shoulder stabilisation. J Bone Joint Surg Br.

[31] Flinkkila T, Hyvonen P, Ohtonen P, Leppilahti J (2010). Arthroscopic Bankart repair: results and risk factors of recurrence of instability. Knee Surg Sports Traumatol Arthrosc.

[32] Imhoff AB, Ansah P, Tischer T, Reiter C, Bartl C, Hench M, Spang JT, Vogt S (2010). Arthroscopic repair of anterior-inferior glenohumeral instability using a portal at the 5:30-o'clock position: analysis of the effects of age, fixation method, and concomitant shoulder injury on surgical outcomes. Am J Sports Med.

[33] Kandziora F, Jager A, Bischof F, Herresthal J, Starker M, Mittlmeier T (2000). Arthroscopic labrum refixation for post-traumatic anterior shoulder instability: suture anchor versus transglenoid fixation technique. Arthroscopy.

[34] Nakagawa S, Hirose T, Tachibana Y, Iuchi R, Mae T (2017). Postoperative recurrence of instability due to new anterior glenoid rim fractures after arthroscopic Bankart repair. Am J Sports Med.

[35] Burkhart SS, De Beer JF (2000). Traumatic glenohumeral bone defects and their relationship to failure of arthroscopic Bankart repairs: significance of the inverted-pear glenoid and the humeral engaging Hill-Sachs lesion. Arthroscopy.

[36] Hayashida K, Yoneda M, Nakagawa S, Okamura K, Fukushima S (1998). Arthroscopic Bankart suture repair for traumatic anterior shoulder instability: analysis of the causes of a recurrence. Arthroscopy.

[37] Ungersbock A, Michel M, Hertel R (1995). Factors influencing the results of a modified Bankart procedure. J Shoulder Elbow Surg.

[38] Walton J, Paxinos A, Tzannes A, Callanan M, Hayes K, Murrell GA (2002). The unstable shoulder in the adolescent athlete. Am J Sports Med.

[39] Bah A, Lateur GM, Kouevidjin BT, Bassinga JYS, Issa M, Jaafar A, Beaudouin E (2018). Chronic anterior shoulder instability with significant Hill-Sachs lesion: arthroscopic Bankart with remplissage versus open Latarjet procedure. Orthop Traumatol Surg Res.

[40] Bhatia S, Saigal A, Frank RM, Bach BR, Cole BJ, Romeo AA, Verma NN (2015). Glenoid diameter is an inaccurate method for percent glenoid bone loss quantification: analysis and techniques for improved accuracy. Arthroscopy.

[41] Lo IK, Parten PM, Burkhart SS (2004). The inverted pear glenoid: an indicator of significant glenoid bone loss. Arthroscopy.

[42] Wang L, Kang Y, Li Y, Wu C, Jiang J, Yu S, Zhao J, Xie G (2021). Dynamic double-sling augmentation prevents anteroinferior translation for recurrent anteroinferior shoulder dislocation with 20% glenoid bone loss: a cadaveric biomechanical study. Arthroscopy.

[43] Itoi E, Lee SB, Berglund LJ, Berge LL, An KN (2000). The effect of a glenoid defect on anteroinferior stability of the shoulder after Bankart repair: a cadaveric study. J Bone Joint Surg Am.

[44] Shaha JS, Cook JB, Song DJ, Rowles DJ, Bottoni CR, Shaha SH, Tokish JM (2015). Redefining “critical” bone loss in shoulder instability: functional outcomes worsen with “subcritical” bone loss. Am J Sports Med.

