# Supplementary material for: Risk factors for recurrence after Bankart repair: a systematic review and meta-analysis
Source: J Orthop Surg Res. 2022 Feb 20;17:113. doi: 10.1186/s13018-022-03011-w (PMC8859902; doi:10.1186/s13018-022-03011-w)
Supplement: Supplementary file 2 — Additional file 2. Other risk factors related recurrent shoulder instability. [file 13018_2022_3011_MOESM2_ESM.doc]

| **Table 13** Other risk factors related recurrent shoulder instability | | |
| --- | --- | --- |
| Risk factor | Studies | Results |
| Number of sutures | Hayashida, Shibata, Flinkkila,van,Boileau | Hayashida (The patients with 5 sutures or fewer had more chance of redislocation  (p＜0.05)); Flinkkila and van (the use of less than 3 suture anchors might increase the chance of a redislo-cation); Shibata and Boileau (the number of suture anchors <4 was significant risk factors for re-dislocation after ABR). |
| Bankart lesion | Ungersbock, Hayashida | Ungersbock (Bankart lesion >50% was significant risk factor); Hayashida (the risk factor  significantly related to recurrence was a type 3 Bankart lesion). |
| Small bone fragment of bony Bankart lesion | Nakagawa | There is significant association between small bone fragment of bony Bankart lesion with  postoperative recurrence of instability(p＜0.05). |
| Capsular tear | Nakagawa | Capsular tear was significant risk factor(p=0.0035). |
| GLAD lesions | Pogorzelski | GLAD lesions were associated with higher rates of failure. |
| Duration of symptoms | Dekker,Hayashida | Dekker (duration of symptoms before presentation (>5 months) was significantly risk factor); Hayashida (duration of symptoms was not an important risk factor). |
| Re-injury within the first year | Shibata | Re-injury within the first year was a risk for re-dislocation after ABR (P < 0.001). |
| Number of episodes | Tamali, Porcellini | There is no association between number of episodes (>3) and recurrent instibility(p=0.49). |
| Mechanism of injury | Flinkkila | Mechanism of injury was not an important risk factor(p=0.47) |
| The time from the first dislocation  to surgery | Porcellini | An interval of more than six months between the first dislocation and surgery was significant risk factor(p＜0.05). |
| ABR, Arthroscopic Bankart repair | | |
